# Enhancing physical, technical, tactical, and cognitive performance in female youth football players using eye-tracking and neurofeedback training: an exploratory study

**DOI:** 10.3389/fpsyg.2026.1835322

**Published:** 2026-06-10

**Authors:** Sílvio A. Carvalho, Pedro Bezerra, José E. Teixeira, Pedro Forte, Rui M. Silva, José M. Cancela-Carral

**Affiliations:** 1 Faculty of Educational Sciences and Sports Sciences, University of Vigo, Pontevedra, Spain; 2 SPRINT—Sport Physical Activity and Health Research & Innovation Center, Viana do Castelo, Portugal; 3 Department of Sports, Higher Institute of Educational Sciences of the Douro, Penafiel, Portugal; 4 School of Sports and Leisure, Polytechnic Institute of Viana do Castelo, Viana do Castelo, Portugal; 5 Department of Sports Science, Polytechnic of Cávado and Ave, Guimarães, Portugal; 6 Department of Sports Science, Polytechnic Institute of Guarda, Guarda, Portugal; 7 CI-ISCE, ISCE Douro, Penafiel, Portugal; 8 Research Center for Active Living and Wellbeing (Livewell), Instituto Politécnico de Bragança, Bragança, Portugal; 9 Department of Sports Science, Instituto Politécnico de Bragança, Bragança, Portugal

**Keywords:** cognitive, eye-tracking, neurofeedback, performance, soccer, youth female

## Abstract

This study aimed to investigate the effects of an intervention combining eye-tracking and neurofeedback training on technical, tactical, and cognitive performance in young female football players. A cross-sectional study was conducted with eight female youth football players (15.6 ± 1.2 years) recruited from a FIFA-certified training centre. Anthropometric measures (body mass, standing height, and seated height) were recorded, and biological maturity was assessed using the Mirwald equation. Performance was evaluated using standardized tests: Loughborough Football Shooting Test (LSST) for technical skill, Yo-Yo Intermittent Recovery Test Level 1 (YYIR1) for endurance, and a system of tactical assessment in football (FUT-SAT) instrument for motor and tactical proficiency. For clarity, we define the measurement moments used throughout this article: baseline assessments are denoted as moment 1 (M1) and post-intervention assessments after the six-week training period as moment 2 (M2). Eye-tracking metrics, decision-making indexes, and neurofeedback parameters were analysed using i-Brain and i-Tracker technologies. Players showed significant improvements in technical and tactical performance, particularly in shooting velocity and offensive unit efficiency, alongside enhanced decision-making stability and defensive coordination. Eye-tracking metrics revealed increased fixation efficiency and reduced revisit counts, indicating more effective visual search strategies. YYIR1 performance improved, suggesting that the intervention did not impair intermittent recovery capacity. The integration of eye-tracking and neurofeedback training demonstrated potential for enhancing cognitive and technical performance in female youth football players. Improvements in shooting accuracy, decision-making speed, and gaze efficiency suggest that these technologies could be valuable additions to traditional training methods.

## 1 Introduction

Female youth football players are increasingly recognized in sports science research due to their physiological and cognitive developmental trajectories, which distinctly influence their technical, tactical, and neurofeedback performance in competitive environments. The literature indicates that female football players show unique motor learning patterns, decision-making processes, and visual scanning behaviours contrasted against male counterparts (Kwabena et al., 2023; Legault and Faubert, 2024; Meng et al., 2019). Such differentiation in development may stem from a variety of factors, including differences in training regimens, cognitive skill acquisition, and hormonal influences that contribute to their distinctive physiological responses during sports engagement (Kirby et al., 2024; Kurka et al., 2014). Notably, technical skills like dribbling, passing, and shooting accuracy are pivotal to their overall performance. Simultaneously, tactical awareness is critical for performance at higher levels of competition, including positioning, spatial awareness, and differentiated strategies for defense, as these elements allow women football players to navigate complex game situations effectively (Buser et al., 2025; Butler et al., 2024).

Furthermore, recent neurofeedback studies have underscored that cognitive processing speed, gaze behaviour, and reaction time serve as crucial determinants of successful performance during games. These aspects are particularly vital in dynamic match situations where quick decisions are often necessary (Aguilar-Navarro et al., 2021; Oldham et al., 2025). The reinforcement of these skills can be achieved through targeted cognitive training interventions, emphasizing the relationship between physical engagement in sport and cognitive processing improvements. Evidence from longitudinal studies suggests that sustained physical activity and the acquisition of sport-specific skills positively influence cognitive functioning, drawing attention to the potential for football training to enhance not only physical capabilities but also neurological agility (Churchill et al., 2021; Gromeier and Schack, 2019).

To thoroughly assess these performance dimensions, a variety of methodologies and instruments have been employed. Technical performance is frequently evaluated through standardized drills such as the Loughborough Football Shooting Test (LSST) and specialized ball control exercises (Legault and Faubert, 2024; Stenling et al., 2014; Stracciolini et al., 2014). Tactical proficiency is often appraised via game simulations paired with advanced movement tracking systems, allowing researchers to observe decision-making in real-time contexts (Benjaminse et al., 2015; Buser et al., 2025). Additionally, neurofeedback, together with cognitive performance evaluations, can be enhanced through innovative technologies, such as gaze-tracking systems and electroencephalography (EEG) monitoring (Başkaya et al., 2023; Moradi et al., 2024). The integration of these methodologies provides a comprehensive framework for understanding how young female players engage with and respond to match dynamics, ultimately informing more effective training strategies (Jaitner and Mess, 2019; Legault and Faubert, 2024).

Recent advancements in sports technology have fostered the emergence of innovative interventions that aim to enhance athletic performance through sophisticated tools like eye-tracking systems and neurofeedback devices, such as i-Brain. Eye-tracking technology facilitates real-time analysis of women football players’ visual attention, fixation patterns, and rapid tracking movements (Mohebi et al., 2021; Stenling et al., 2014). The resultant insights can be instrumental in refining decision-making processes and gaze strategies during competitive play. Equally, i-Brain, a cutting-edge neurofeedback training system, utilizes EEG-based biofeedback mechanisms that target cognitive control, concentration, and response inhibition (Denham, 2022; Gürsoy and Canli, 2021). Together, these technological advancements highlight the promise of combining traditional training methods with modern cognitive training frameworks, thereby leading to significant improvements not only in technical execution but also in tactical acumen and psychological resilience for female football players (Mohebi et al., 2021).

Despite these developments, research exploring the combined effects of eye-tracking and neurofeedback training on female youth football players remains limited. Most existing studies focus on male football players, leaving a gap in understanding how these technologies can specifically benefit female players. Although male and female athletes share many fundamental technical skills, there are sex-specific differences in maturation, hormonal profiles and training histories that may influence cognitive and motor adaptations to technology-assisted training. Recent neuroimaging work demonstrates that complex visuo-motor learning induces distinct patterns of white-matter neuroplasticity in women versus men (Kirby et al., 2024). These findings highlight the importance of studying female populations rather than generalizing male-derived data. This study aims to investigate the impact of an intervention combining eye-tracking and neurofeedback training on technical, tactical, and cognitive performance in young female football players. In particular, the research addresses the neglected topic of how female youth players adapt to combined perceptual-cognitive training and provides a clear hypothesis that after a six-week intervention the athletes will display significant improvements in shooting accuracy, decision-making speed, and gaze efficiency relative to baseline. We hypothesize that integrating these technologies into training programs will lead to significant improvements in shooting accuracy, decision-making speed, and gaze efficiency, ultimately enhancing overall game performance.

## 2 Materials and methods

### 2.1 Design and participants

**Figure 1:**
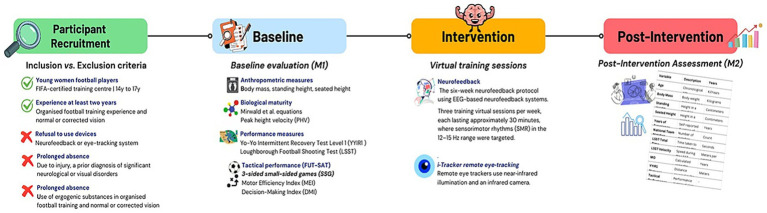
Intervention diagram.

This study followed a cross-sectional experimental design conducted over a six-week period to examine the effects of combined eye-tracking and neurofeedback training on technical, tactical, and cognitive performance in female youth football players. Eight athletes aged 14–17 years were recruited from a FIFA-certified training centre and assessed at two time points: baseline (M1) and post-intervention (M2) (Figure 1).

**Figure 2:**
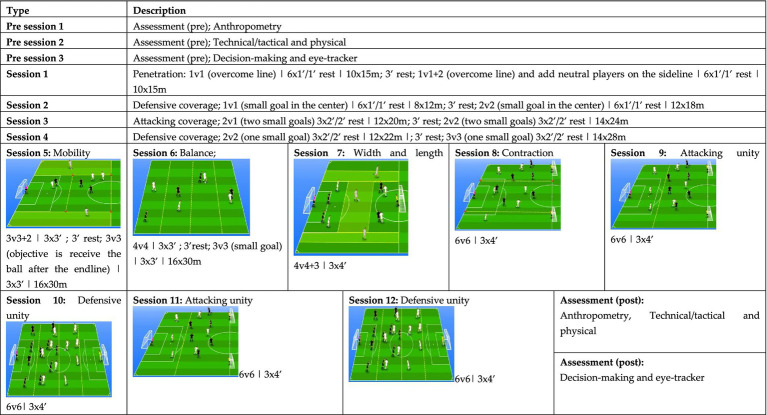
Overview of standardized tests employed in the study, including the detailed intervention schedule and assessment points.

Anthropometric variables, biological maturity, and football-specific performance were measured using validated protocols, including the Loughborough Football Shooting Test (LSST), Yo-Yo Intermittent Recovery Test Level 1 (YYIR1), and the FUT-SAT framework for tactical behaviour (Figure 2). The intervention integrated i-Tracker remote eye-tracking and i-Brain EEG-based neurofeedback sessions administered three times per week, targeting gaze efficiency, sensorimotor rhythm modulation, and decision-making processes. Eye-tracking metrics, neurofeedback performance indices, technical execution, and tactical decision-making outcomes were compared between baseline (M1) and post-intervention (M2) to quantify cognitive-perceptual and motor changes. Ethical approval and informed consent were obtained prior to participation, and inclusion/exclusion criteria ensured athlete safety and data quality throughout the intervention.

The sample consisted in eight youth women football players (15.6 ± 1.2 years). The mean age of participants was 15.6 years, with a standard deviation of 1.2 years. Participants were recruited from local A FIFA-certified women’s football training centre. Ethical considerations were addressed, and informed consent was obtained from all participants and guardians. Data were analysed to assess changes in neurophysiological activity, skill performance, and decision-making quality over the intervention period. To contextualize the sample, we now report players’ positional roles, competitive experience and training practices. The cohort comprised one goalkeeper, three defenders, two midfielders and two forwards. Participants had an average of 6.7 ± 1.4 years of organized competitive experience and had been affiliated with their current club for 3.2 ± 1.1 years. The weekly training schedule consisted of four on-field technical/tactical sessions and two strength and conditioning sessions with a total duration of 90 min per session. All women have signed an informed consent form. All the players presented their free and informed consent signed by themselves or, if they were minors, by their legal representative. The ethical principles outlined in the Declaration of Helsinki were followed with the CECSVS2023/11/viii ethical committee approval.

To ensure the internal validity and safety of the study, we specified inclusion and exclusion criteria that were not clearly detailed in the original version. Only female athletes aged between 14 and 17 years, with at least 2 years of organized football training experience and normal or corrected vision, were included. Participants could not have a history of neurological or orthopaedic injuries, nor be taking psychotropic medication. The athletes had to be available to attend all assessment and intervention sessions throughout the six-week study period. Exclusion criteria included refusal to use the neurofeedback device or the eye-tracking system, prolonged absence due to injury, a prior diagnosis of significant neurological or visual disorders, and the use of ergogenic substances.

### 2.2 Anthropometric measures

Anthropometric measurements (body mass, standing height, seated height) were conducted by trained personnel to ensure accuracy. The following outlines the recommended materials and procedures for measuring body mass, standing height, and seated height, based on recent guidelines and best practices (Abreu et al., 2022; Aristizabal et al., 2007). A digital floor scale with a precision of ±0.1 kg is recommended for measuring body mass. A wall-mounted stadiometer is used for measuring standing height. The floor should be level and hard to ensure accurate measurements. A flexible, non-stretchable tape measure is essential for measuring seated height and other body dimensions. Standardized procedures should be followed to ensure accuracy. In this study body mass was measured using a portable digital scale (Seca 874, Seca GmbH, Hamburg, Germany) with a 0.1 kg resolution. Standing height was assessed with a wall-mounted stadiometer (Holtain, Crymych, United Kingdom) accurate to 1 mm, and seated height was determined using a flexible metric tape (Lufkin W606, Cooper Hand Tools, United States). All instruments were calibrated prior to testing, and participants were measured barefoot and wearing light sport clothing to minimize measurement error.

### 2.3 Biological maturity

The maturity offset (MO) was calculated using established predictive equations, which have been validated in youth populations. The Mirwald et al. equations are widely utilized to estimate maturity offset, representing the number of years a youth is from reaching their peak height velocity (PHV) (Malina et al., 2019). Standing height should be measured with the individual standing upright, without shoes, ensuring the head is in the Frankfort horizontal plane. Sitting height requires the individual to sit on a flat surface with a straight back, measuring the distance from the sitting surface to the top of the head. Body mass should be recorded with the individual in light clothing and without shoes. The MO was calculated as follow equation: MO (years) = −9.376 + (0.0001882 × (Leg Length × Sitting Height)) + (0.0022 × (Age × Leg Length)) + (0.005841 × (Age × Sitting Height)) + (0.02416 × A negative) MO indicates the number of years before PHV, while a positive value signifies the number of years after PHV. Specifically, maturity timing was estimated by *z* scores: higher than 0.5 (early status); between −0.5 and +0.5 (average maturity timing; this means the players were considered as average in their maturity stages); and below −0.5 (late maturity timing) (Malina et al., 2019; Teixeira et al., 2022a).

### 2.4 Performance measures

Performance metrics will be assessed through standardized tests, including the LSST and YYIR1, which are recognized for their reliability in evaluating athletic performance (Serpiello et al., 2017). The LSST and YYIR1 have established assessments for evaluating football-specific skills and endurance, respectively. Their application in women’s football has been documented, providing insights into performance metrics. Tactical performance was measured by a system of tactical assessment in football (FUT-SAT) instrument, considering correct and incorrect actions for motor efficiency and decision-making (Silva et al., 2014).

### 2.5 Loughborough Football Shooting Test

The LSST is designed to assess a player’s shooting accuracy and proficiency under conditions that mimic match play. During the test, players are required to execute a series of shots on goal from designated positions, aiming to hit specific targets within the goal area. The test is structured to evaluate both precision and decision-making speed. Performance is quantified based on the number of successful target hits and the time taken to complete the sequence. This test has been validated for use with female players, demonstrating reliability in distinguishing skill levels among participants (Ali et al., 2008; Serpiello et al., 2017).

### 2.6 Yo-Yo Intermittent Recovery Test Level 1

The YYIR1 assesses an women football player’s capacity to perform repeated high-intensity aerobic work with brief recovery periods, reflecting the intermittent nature of football. The test involves repeated 2 × 20-meter shuttle runs at increasing speeds, interspersed with short recovery intervals. The total distance covered before exhaustion serves as the performance indicator. While the YYIR1 is a common measure of fitness in football, studies have indicated that it may not accurately assess or estimate VO₂ max in female football players, suggesting the need for gender-specific evaluation protocols (Bangsbo et al., 2008; Martínez-Lagunas and Hartmann, 2014).

### 2.7 Tactical performance (FUT-SAT)

Tactical performance was analysed using a FUT-SAT-based instrument, which quantified correct and incorrect actions to assess motor efficiency and decision-making in 3-sided small-sided games (SSG) (Matos et al., 2023). The training programme consisting of 12 sessions of SSGs. The sessions were specifically structured to develop different offensive and defensive tactical principles, ensuring pedagogical progression and increasing situational complexity. Each session focused on a particular tactical principle, specifically the penetration, defensive coverage, offensive coverage, mobility, balance, offensive unity, and defensive unity. It was operationalised through reduced tasks (1v1, 2v2, 3v3, 4v4, 6v6). Manipulations of playing space, number of players, target positioning, and finishing conditions were used to amplify the players’ perception, decision-making, and tactical adaptability.

The SSG intervention consisted of 12 structured training sessions, each designed to elicit specific tactical principles through manipulation of numerical relations, field dimensions, and scoring constraints. The first session targeted penetration, beginning with 1v1 scenarios aimed at overcoming a defensive line (6 × 1 min, 1-min rest, 10 × 15 m), followed by a progressed 1v1 + 2 neutrals format using the same temporal structure to increase decision-making complexity. Defensive coverage was trained through 1v1 tasks involving a central mini goal (6 × 1 min, 8 × 12 m), followed by 2v2 on a larger pitch (12 × 18 m), encouraging coordinated defensive behaviour. Attacking coverage was addressed through 2v1 situations with two mini-goals (3 × 2 min, 2-min rest, 12 × 20 m) and progressed to 2v2 (14 × 24 m), promoting offensive support and exploitation of space. A second defensive coverage session included 2v2 (12 × 22 m) and 3v3 (14 × 28 m) tasks focusing on compactness and defensive synchronization. Mobility was trained via 3v3 + 2 neutrals (3 × 3 min) to encourage dynamic positional play, followed by 3v3 with the objective of receiving beyond the endline (16 × 30 m). Balance was trained through 4v4 (3 × 3 min) and 3v3 with mini goals (16 × 30 m), focusing on maintaining structural stability. Width and length were developed using 4v4 + 3 neutrals (3 × 4 min), which promoted expansion of play and exploitation of pitch dimensions. The final sessions addressed contraction, attacking unity, and defensive unity through repeated 6v6 formats (3 × 4 min), designed to simulate match-like density and reinforce coordinated collective behaviour in both offensive and defensive phases.

The final evaluated variables were: motor efficiency index (MEI) encompasses various dimensions of tactical performance, including MEI penetration, which measures the ability to make effective decisions for breaking through defensive lines; MEI offensive coverage, which evaluates support for the offensive unit through decision-making and positioning; MEI depth mobility, assessing off-ball movement to optimize depth in attacks; MEI space, measuring the creation and utilization of space; MEI offensive unit, which reflects overall efficiency in offensive decision-making; MEI delay, which indicates effectiveness in disrupting opponent transitions; MEI defensive coverage, evaluating decisions that bolster defensive structure; MEI balance, assessing maintenance of tactical equilibrium in both attack and defense; MEI concentration, reflecting focus in decision-making; and MEI defensive unit, capturing overall defensive efficiency. Similarly, decision-making (DM) metrics include DM pen, evaluating decision-making related to penetration strategies; DM OC (offensive coverage), reflecting support for the offensive unit; DM mobility, assessing movement-related positioning; DM space, measuring spatial efficiency; DM offensive unit, a composite score for offensive decision-making; DM delay, gauging efficiency in delaying transitions; DM DC (defensive coverage), evaluating defensive decisions; DM balance, reflecting positional equilibrium; DM concentration, highlighting critical focus in decision-making; and DM defensive unit, summarizing defensive decision-making performance. Additional variables include the decision-making index (DMI), which combines tactical decision-making scores, and performance, representing overall tactical and technical efficiency.

To implement the tactical assessment, the players participated in 3 × 3 small-sided games designed to elicit repeated offensive and defensive actions. Each game lasted 8 min and was played on a 36 × 27 m pitch with small goals and no goalkeepers, replicating typical youth training. Sessions were video recorded and later coded using the system of FUT-SAT (Costa et al., 2011; Silva et al., 2014). This observational tool classifies player actions according to 10 core tactical principles—penetration, offensive coverage, depth mobility, width and length, offensive unity, delay, defensive coverage, balance, concentration, and defensive unity—and has been validated in youth soccer (Silva et al., 2014). For each action, trained coders judged whether the decision was correct or incorrect for the relevant principle, and the frequency of correct decisions was used to compute the MEI and DM metrics (Costa et al., 2011; Matos et al., 2023).

### 2.8 Neurofeedback

The six-week neurofeedback protocol for youth female football players focused on enhancing motor skills and tactical decision-making through targeted interventions using EEG-based neurofeedback systems. Participants engaged in three sessions per week, each lasting approximately 30 min, where sensorimotor rhythms (SMR) in the 12–15 Hz range were targeted. Training included visualization of football-specific scenarios to improve motor imagery and decision-making. Sessions began with baseline assessments to establish individual brain activity patterns, followed by real-time neurofeedback tasks using virtual environments that simulated game-specific challenges, such as accurate passing, shooting, and defensive positioning. Feedback was provided visually through a computer screen to guide participants in modulating SMR activity. The protocol incorporated progressive difficulty, starting with static tasks and advancing to dynamic challenges mimicking competitive game scenarios. Researchers ensured individualized adjustments based on performance data to optimize neuroplasticity and motor skill development. This protocol was grounded in evidence from motor imagery and neurofeedback studies, showing improved cortical and corticospinal tract excitability (Komarudin et al., 2021; Neurocare Group, 2025).

The assessed variables were power, representing the participant’s ability to make quick tactical decisions or demonstrate technical actions under time constraints. Accuracy indicates how precise the participant’s decisions/actions were in alignment with the optimal tactical and technical responses. Goals, percentage of successful decisions/actions that directly or indirectly aligned with achieving a goal (e.g., scoring, advancing play effectively). Power difficulty level (DL), the complexity of tactical scenarios requiring quick decision-making or technical execution. Higher values suggest greater difficulty in decision-making under pressure. Accuracy DL, the complexity or ambiguity in scenarios requiring precise decisions or actions. Higher values imply more challenging situations where multiple options may seem viable. Power sum, aggregated or weighted contribution of tactical decision-making speed and technical execution across all tasks, potentially normalized to account for difficulty. Accuracy sum, aggregated or weighted contribution of tactical and technical precision across all tasks, also normalized for difficulty.

#### 2.8.1 Technical setup and signal processing

The neurofeedback training was delivered via a ProComp Infiniti^™^ encoder (Thought Technology Ltd., Montreal, Canada), an eight-channel multi-modality device widely used for biofeedback and neurofeedback. The first two sensor channels of this encoder provide a high sampling rate of 2048 samples per second, while the remaining six channels are sampled at 256 samples per second (Thought Technology Ltd., 2023). Silver/silver-chloride electrodes were placed over C3, Cz and C4 according to the international 10–20 system, with reference electrodes positioned at the mastoid sites (T7/T8), to target the sensorimotor rhythm. Electrode impedance was kept below 5 kΩ. Raw EEG signals were filtered online with a 1–30 Hz band-pass filter, and ocular and muscle artefacts were identified and removed using independent component analysis before SMR power was calculated. Visual feedback was presented on a computer screen as moving bars or game elements that increased when SMR amplitude at 12–15 Hz exceeded a dynamically adjusted threshold. Participants were instructed to relax and focus on football-specific scenes during training. Each session began with a five-minute baseline recording used to calibrate the feedback threshold and concluded with a cool-down period. The combination of high sampling fidelity and real-time processing provided accurate and responsive feedback to facilitate cortical self-regulation.

### 2.9 i-Tracker remote eye-tracking

Visual search strategies were recorded using an i-Tracker remote eye-tracking system. Remote eye trackers use near-infrared illumination and an infrared camera to monitor pupil position and corneal reflections, allowing computation of the gaze point without requiring head restraint (Duchowski, 2013; Kooiker et al., 2016). Key eye-movement parameters include fixations, periods when the eyes remain relatively still to acquire visual information, and saccades, rapid movements that shift gaze between fixations (Eyeware, 2022). Derived variables calculated in this study were time to first fixation (TTFF), dwell count and dwell time, revisit count (the number of returns to an area of interest), fixation count, first and last fixation duration, fixation dispersion, saccade count, average saccade duration and amplitude, peak saccade velocity and acceleration, and the proportion of time spent fixating. These metrics quantify visual attention and scanning behaviour during technical drills and small-sided games. Remote eye-tracking has demonstrated good agreement with standard visual function assessments and is considered a valid method for evaluating oculomotor in football performance (Aksum et al., 2021; Casanova et al., 2022).

#### 2.9.1 Instrumentation and calibration

The eye-tracking data were collected using a remote TRACKPixx/mini system (VPixx Technologies Inc., Saint-Bruno, Canada). This device employs dark-pupil binocular tracking with a sampling rate of 120 Hz, spatial accuracy of approximately 0.5° and a head movement box of about 20 × 5 × 10 cm (VPixx Technologies, 2024). During testing participants sat 60 cm from the display and completed a five-point calibration followed by a validation routine; calibrations with mean error <0.5° were accepted, and recalibration was performed whenever errors exceeded this criterion. Gaze data were pre-processed by applying a Savitzky–Golay filter to reduce noise. A velocity-based saccade detection algorithm with a 30°·s − 1 threshold identified saccades, and fixations were defined using a dispersion threshold of 1° sustained for at least 100 ms. Areas of interest (AOIs) corresponding to the ball, teammates, opponents and goal were manually delineated on the video recordings, and dwell times and counts were computed relative to these AOIs. These methodological details facilitate reproducibility and were absent from the original submission.

### 2.10 Outcomes

The variables analyzed were age, body mass, standing height, seated height, years of experience, national team matches, LSST total time, LSST velocity, MO, YYIR1 distance, and performance metrics (M1 and M2) correlate with each other (Table 1). Moment 1 (M1) refers to the baseline assessment conducted before the intervention, whereas moment 2 (M2) corresponds to the follow-up evaluation immediately after 6 weeks of eye-tracking and neurofeedback training. Defining these moments clarifies the temporal framework for interpreting longitudinal changes.

**Table 1:** Descriptive characteristics of participants at the chronological and functional levels.

Variable	Description	Years
Age	Chronological age	KilYears
Body mass	Body weight measured on a calibrated scale	Kilograms
Standing height	Height in a standing position measured with a stadiometer	Centimeters
Seated height	Height in a seated position measured with a stadiometer	Contimeters
Years of experience	Self-reported duration of competitive sports participation	Years
National team matches	Number of matches played at the national level	Count
LSST total time	Time taken to complete the Loughborough Football Shooting Test	Seconds
LSST velocity	Speed during the LSST calculated from performance	Meters per second
MO	Calculated using the Mirwald equation based on physical parameters	Years
YYIR1 distance	Distance covered in the Yo-Yo Intermittent Recovery Test Level 1	Meters
Tactical performance	Performance metrics during the first measurement phase	-

### 2.11 Statistical analysis

Given the small sample size (*n* = 8), assumptions of normality and homoscedasticity were assessed using Shapiro–Wilk and Levene tests. When *p*-values were above 0.05, skewness (<2) and kurtosis (<7) statistics justified the use of parametric tests. *A priori* power analysis using G*Power 3.1 suggested that at least 10 participants would be required to detect a large effect size (*d* = 0.8) with 80% power in a within-subjects design; due to the limited availability of eligible players, our sample comprised eight athletes, and the present study should therefore be considered an exploratory pilot. Descriptive statistics, including means, medians, standard deviations, and ranges, were calculated for all variables to summarize the data effectively. To compare numerical variables, mean values and standard deviations for two moments (M1 and M2) along with their percentual changes (Δ%). To compare numerical variables between women football players grouped by their dominant foot, independent samples *t*-tests were performed. The significance level was set at *p* < 0.05, indicating that any *p*-value below this threshold would be considered statistically significant. Effect sizes were calculated using Cohen’s *d* to assess the practical significance of the differences observed, with values interpreted as small (*d* > 0.20), moderate (*d* > 0.50), and large (*d* > 0.80) (Aschwanden and Nguyen, 2018). ANOVA was employed to explore variations across playing positions, with the F-statistic and corresponding p-values reported and the Bonferroni correction was used. Effect sizes for ANOVA were calculated using eta squared (*η*^2^) and deemed as: (i) without effect if 0 < *η*^2^ < 0.04; (ii) minimum if 0.04 < *η*^2^ < 0.25; (iii) moderate if 0.25 < *η*^2^ < 0.64 and; (iv) strong if *η*^2^ > 0.64 (Hopkins et al., 2009). Pearson correlation coefficients were calculated to examine the relationships between numerical variables. Correlation coefficients were interpreted as follows: weak (0.1 to 0.3), moderate (0.3 to 0.5), and strong (0.5 to 1.0) (Altman and Krzywinski, 2015). A significance level of *p* < 0.05 was also applied to the correlation analysis to determine the statistical significance of the relationships. The statistical analysis for this study was conducted using IBM SPSS software v.26.

## 3 Results

Table 2 presents a detailed summary of the performance metrics across multiple variables, comparing the mean values and standard deviations for two moments (M1 and M2) along with their percentual changes. These metrics span different areas, including physical performance, decision-making, gaze behaviour, and motor efficiency. For the YYIR1 test, the results indicate a positive improvement in intermittent recovery capacity, with an increase of 13.94%. Similarly, variables such as “LSST_Shot Points (average of 10 shots)” and “MEI offensive unit” demonstrated significant positive changes, with percentual variations of 7.21 and 64.81%, respectively. Other variables, like “Dwell count” and “Revisit count (gaze dwells),” also showed notable increases, reflecting improvements in gaze-related performance measures. In contrast, some metrics revealed declines, such as “DM defensive unit” (−39.19%) and “TTFF (AOI)” (−34.52%), indicating potential areas of reduced efficiency. Variables related to saccade behaviour, such as “Duration of average saccade,” showed modest increases (3.03%), while others like “Amplitude of average saccade” decreased (−5.33%), reflecting varied adaptations.

**Table 2:** Descriptive statistics for technical, tactical and gaze-related variables across two measurement moments (M1 and M2).

Variable	M1 mean ± SD	M2 mean ± SD	Δ %	Variable	M1 mean ± SD	M2 mean ± SD	Δ %	Variable	M1 mean ± SD	M2 mean ± SD	Δ %
YYIR1	416.00 ± 224.46	474.00 ± 243.66	13.94	MEI depth mobility	0.662 ± 0.132	0.687 ± 0.161	3.78	Revisit count (fixation dwells)	0.559 ± 0.773	0.927 ± 1.07	65.83
LSST_Shot points (average of 10 shots)	2.345 ± 0.532	2.514 ± 0.803	7.21	MEI space	0.635 ± 0.09	0.637 ± 0.167	0.31	Fixation count	4.194 ± 3.208	4.672 ± 3.496	11.4
LSST_Total time (s) mean	10.484 ± 1.539	10.016 ± 0.575	−4.46	MEI offensive unit	0.466 ± 0.088	0.768 ± 0.273	64.81	TTFF sequence	1.667 ± 0.727	1.753 ± 0.926	5.16
LSST_Shot velocity (km/h)_Mean	30.058 ± 9.573	26.306 ± 10.801	−12.48	MEI	0.624 ± 0.065	0.675 ± 0.065	8.17	TTFF (AOI)	1207.502 ± 1495.154	790.677 ± 1270.411	−34.52
DM delay	0.722 ± 0.140	0.681 ± 0.154	−5.68	Performance	0.672 ± 0.036	0.749 ± 0.096	11.46	TTFF (parent)	7625.28 ± 2356.81	7201.717 ± 2090.673	−5.55
DM DC	0.877 ± 0.107	0.594 ± 0.233	−32.27	Start	437381.69 ± 245436.473	484578.097 ± 282753.111	10.79	TTFF (max.)	8555.398 ± 2841.051	7908.008 ± 2667.55	−7.57
DM balance	0.86 ± 0.139	0.69 ± 0.169	−19.77	Duration (ms)	7591.548 ± 2962.605	7590.686 ± 2953.716	−0.01	Dwell time (fixation, ms)	3106.127 ± 1931.007	3168.656 ± 1880.36	2.01
DM concentration	0.784 ± 0.188	0.691 ± 0.228	−11.86	Parent start	432569.536 ± 245938.445	479765.944 ± 282991.092	10.91	Dwell time (fixation, %)	51.44 ± 31.785	52.585 ± 30.6	2.23
DM defensive unit	0.842 ± 0.123	0.512 ± 0.149	−39.19	Parent duration	12403.703 ± 1624.641	12402.908 ± 1619.04	−0.01	Duration of average fixation	906.223 ± 624.782	855.547 ± 702.947	−5.59
DM pen	0.783 ± 0.203	0.672 ± 0.199	−14.18	Duration (%)	48.789 ± 6.541	48.784 ± 6.514	−0.01	First fixation duration	928.725 ± 906.212	795.664 ± 849.483	−14.33
DM OC	0.648 ± 0.119	0.761 ± 0.206	17.44	Valid data	98.001 ± 4.775	98.402 ± 3.655	0.41	Last fixation duration	997.879 ± 750.393	967.573 ± 1007.753	−3.04
DM mobility	0.637 ± 0.076	0.748 ± 0.195	17.43	Dwell count	2.301 ± 1.309	2.694 ± 1.531	17.08	Dispersion of average fixation	0.428 ± 0.27	0.389 ± 0.24	−9.11
DM space	0.859 ± 0.087	0.708 ± 0.152	−17.58	Revisit count (gaze dwells)	1.301 ± 1.309	1.694 ± 1.531	30.21	Dwells with saccades	1.42 ± 0.609	1.455 ± 0.729	2.46
DM offensive unit	0.532 ± 0.176	0.788 ± 0.218	48.12	Hit sequence	1.753 ± 0.747	1.857 ± 0.937	5.93	Saccade count	4.94 ± 3.119	5.064 ± 3.945	2.51
DMI	0.741 ± 0.051	0.659 ± 0.107	−11.07	Hit time (AOI)	1390.303 ± 1639.934	901.958 ± 1340.798	−35.13	Entry saccade onset (AOI)	1667.991 ± 1576.271	1465.005 ± 1416.477	−12.17
MEI delay	0.663 ± 0.175	0.666 ± 0.229	0.45	Hit time (parent)	7808.08 ± 2460.0	7311.919 ± 2121.899	−6.35	Entry saccade onset (parent)	8087.415 ± 2479.48	7953.101 ± 2340.993	−1.66
MEI defensive coverage	0.55 ± 0.194	0.625 ± 0.332	13.64	Dwell time (gaze, ms)	2536.712 ± 2198.475	2574.598 ± 2098.194	1.49	Duration of average saccade	34.485 ± 13.098	35.529 ± 14.957	3.03
MEI balance	0.592 ± 0.181	0.732 ± 0.228	23.65	Dwell time (gaze, %)	41.963 ± 36.175	42.732 ± 34.607	1.83	Amplitude of average saccade	3.508 ± 1.976	3.321 ± 2.045	−5.33
MEI concentration	0.576 ± 0.163	0.651 ± 0.229	13.02	First dwell duration	1258.772 ± 1699.239	1347.369 ± 1910.232	7.04	Peak velocity of average saccade	81.541 ± 42.122	81.996 ± 45.322	0.56
MEI defensive unit	0.544 ± 0.146	0.712 ± 0.266	30.88	Last dwell duration	1510.025 ± 1825.676	1529.425 ± 1985.582	1.28	Peak acceleration of average saccade	3046.855 ± 1866.792	3002.176 ± 2019.23	−1.47
MEI penetration	0.558 ± 0.161	0.61 ± 0.197	9.32	Skip count	0.0 ± 0.0	0.009 ± 0.093	N/A	Peak deceleration of average saccade	−2467.258 ± 1609.431	−2717.616 ± 2204.35	10.15
MEI offensive coverage	0.62 ± 0.193	0.722 ± 0.26	16.45	Dwells with fixations	1.559 ± 0.773	1.927 ± 1.07	23.6	Direction of average saccade	184.85 ± 61.942	182.598 ± 71.914	−1.22

YYIR1, Yo-Yo Intermittent Recovery Test Level 1; LSST, Loughborough Football Shooting Test; MEI, motor efficiency index; DM, decision-making metric; DMI, decision-making index; TTFF, time to first fixation; AOI, area of interest. MEI variables represent tactical efficiency in penetration, offensive coverage, depth mobility, space creation, offensive unit, delay, defensive coverage, balance, concentration, and defensive unit. DM variables represent decision-making accuracy for the same principles. Eye-tracking metrics include fixation and saccade parameters quantifying gaze behaviour. M1: first evaluation stage (baseline); M2: second evaluation stage (post-Intervention).

**Figure 3:**
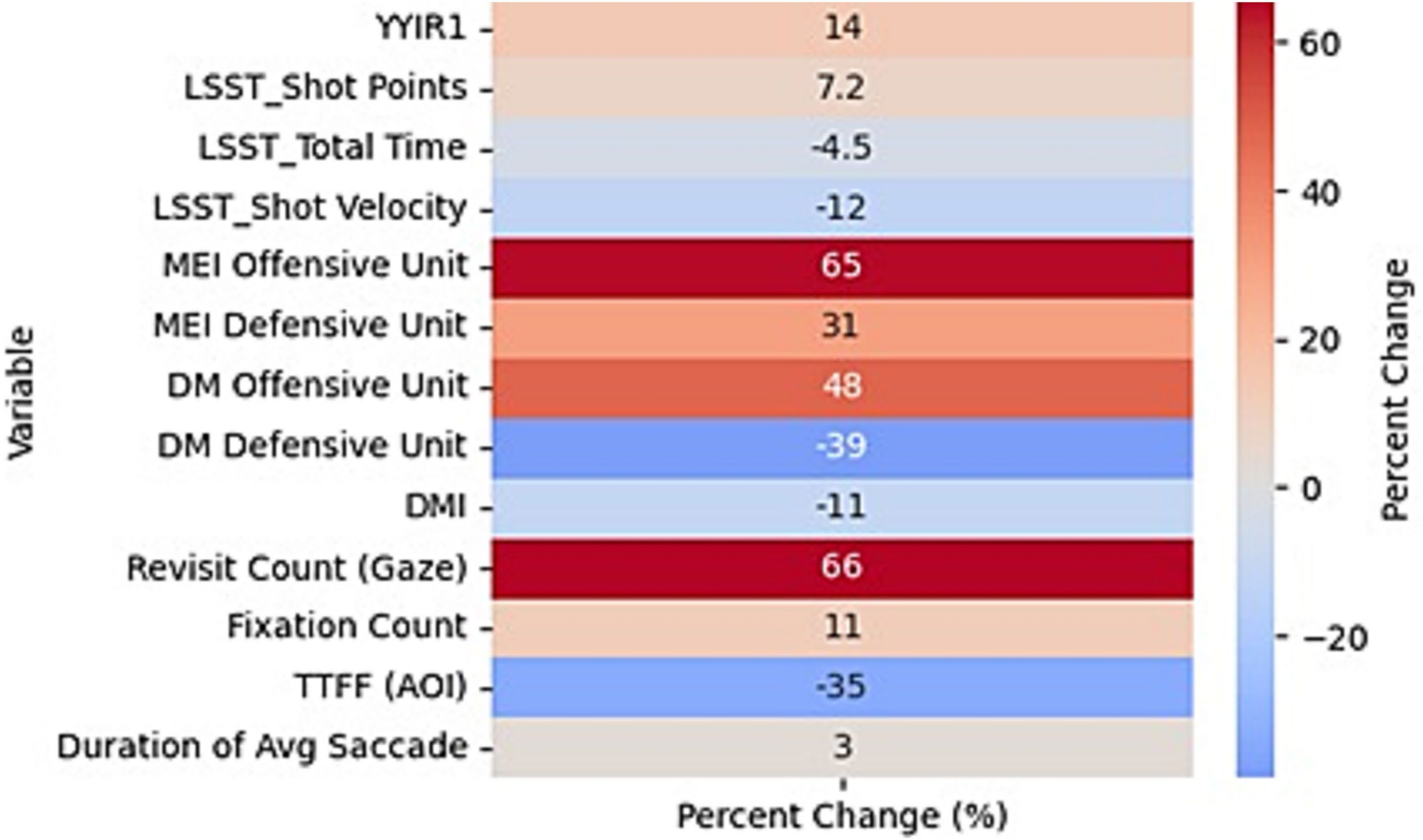
A heatmap illustrating the percentage change (Δ%) of selected technical, tactical and gaze variables between baseline (M1) and post‑intervention (M2) measurements. Red shades denote improvements, whereas blue shades indicate declines.

A heatmap illustrating the percentage change (Δ%) of selected technical, tactical and gaze variables between baseline (M1) and post-intervention (M2) measurements is presented in Figure 3. Red shades denote improvements, whereas blue shades indicate declines.

Table 3 reveals the technical and tactical variables comparisons between moments. In the YYIR1 test, there was a significant increase in performance (*t* = −4.78, *p* < 0.001, *d* = −0.16), indicating an improvement in intermittent recovery capacity. For the LSST variables, shooting speed (k/h), average showed a significant improvement (*t* = 3.12, *p* = 0.006, *d* = −1.35). Interestingly, shooting points (average of 10 shots), average showed no significant change (*t* = −1.70, *p* = 0.106, *d* = −0.01). Among the MEI metrics, offensive unit showed a significant difference (*t* = −4.40, *p* < 0.001, *d* = 0.40), while other metrics such as MEI balance (*t* = −2.07, *p* = 0.054, *d* = 0.73) and MEI concentration (*t* = −1.08, *p* = 0.294, *d* = −0.91) showed non-significant changes. The MEI defensive unit showed a notable improvement (*t* = −2.50, *p* = 0.023, *d* = 0.18). For the DM metric, DM balance (*t* = 4.03, *p* < 0.001, *d* = 2.49) and DM defensive drive (*t* = 7.63, *p* < 0.001, *d* = 2.07) showed substantial positive changes, while DM offensive drive showed a significant decline (*t* = −3.84, *p* = 0.001, d = −0.64). DM delay (*t* = 0.75, *p* = 0.464, *d* = 1.89) showed no significant changes. Regarding gaze-related variables, ‘Revisit count (gaze dwells) showed a significant decrease (*t* = −2.85, *p* = 0.005, *d* = −0.28), as did dwell count (*t* = −2.85, *p* = 0.005, *d* = −0.28). However, metrics such as waiting time (gaze, ms) and waiting time (fixation, ms) showed no significant differences. Finally, for fixation-related metrics, the changes were generally non-significant, as seen in duration of first fixation (*t* = 1.40, *p* = 0.164, *d* = 0.15) and duration of last fixation (*t* = 0.38, *p* = 0.704, *d* = 0.03). Similar trends were observed for the jerk metrics, with most variables such as AOI (*t* = 0.70, *p* = 0.491, *d* = 0.14) showing no significant differences.

**Table 3:** Statistical comparisons between measurement moments for selected variables using *t*-tests and Cohen’s *d* effect sizes.

Variable	*t*	*p*	*d*
YYIR1 (m)	−4.78	<0.001	−0.16
LSST_Shooting velocity (k/h)	3.12	0.006	−1.35
MEI offensive unit	−4.40	<0.001	0.40
MEI defensive unit	−2.50	0.023	0.18
MEI	−2.24	0.039	0.97
DM OC	−2.21	0.042	0.34
DM mobility	−2.15	0.046	1.80
DM space	3.08	0.007	0.60
DM offensive unit	−3.84	0.001	−0.64
DM DC	4.12	<0.001	2.02
DM balance	4.03	<0.001	2.49
DM defensive unit	7.63	<0.001	2.07
DMI	2.52	0.022	1.71
Total performance index	−2.71	0.015	0.38
Start	−2.36	0.019	−0.18
Parent start	−2.04	0.043	−0.18
Dwell count	−2.85	0.005	−0.28
Revisit count (gaze dwells)	−2.85	0.005	−0.28
Hit time (AOI)	2.85	0.005	0.33
Skip count	−5.86	<0.001	−0.13
Dwells with fixations	−4.49	<0.001	−0.39
Revisit count (fixation dwells)	−4.49	<0.001	−0.39
TTFF (AOI)	2.67	0.009	0.30
TTFF (parent)	1.72	0.089	0.19
TTFF (max.)	2.44	0.016	0.24

YYIR1, Yo-Yo Intermittent Recovery Test Level 1; LSST, Loughborough Football Shooting Test; MEI, motor efficiency index; DM, decision-making metric; DMI, decision-making index; TTFF, time to first fixation; AOI, area of interest. All *p*-values <0.05 indicate statistical significance; *d* = Cohen’s effect size.

The analysis revealed significant main effects and interactions for variables with statistical significance are presented in Table 4. The main effect of group was significant for multiple measures, indicating differences between the intervention and control groups. For instance, in LSPT_Tempo S/penalties (s), the main effect of group was significant (*F* = 18.42, *p* < 0.001, *η*^2^ = 0.34, large), while the effect of moment and the moment × group interaction were small or negligible (*η*^2^ = 0.02 and 0.001, respectively). Similarly, in LSPT_Tempo Total (S/ + C/ Penalties), group showed a significant effect (*F* = 12.85, *p* < 0.001, *η*^2^ = 0.29, large), while the Moment × Group interaction remained negligible. For LSST_Tempo Total (s), the main effect of moment was not significant (*F* = 0.59, *p* = 0.449, *η*^2^ = 0.02, small), and group had a large effect (*F* = 18.42, *p* < 0.001, *η*^2^ = 0.34, large), suggesting notable differences between groups. However, interaction effects were generally small or negligible. In performance-related variables, a significant effect of group was observed in M1_Performance (*F* = 7.53, *p* = 0.001, *η*^2^ = 0.19, large), while the effects of moment and the moment × group interaction were minimal (*η*^2^ < 0.02). This pattern suggests that the intervention group demonstrated notable improvements compared to the control group. For decision-making variables, M1_DMI showed a medium effect for group (*F* = 5.25, *p* = 0.021, *η*^2^ = 0.15, medium), but no significant interaction (*p* = 0.735). Similarly, in M1_DM balance, the effect of group was significant (*F* = 4.62, *p* = 0.037, *η*^2^ = 0.13, medium), indicating better performance in the intervention group.

**Table 4:** Two-way ANOVA results comparing group and moment effects for technical and tactical variables.

Variable	Source of variation	*F*	*p*	*η* ^2^	Qualitative ES
LSST_Shot velocity (km/h)_Mean	Group	65.02	<0.001	0.64	Large
Offensive unit	Moment	18.85	<0.001	0.37	Large
MEI concentration	Group	11.15	0.002	0.26	Large
MEI	Moment	6.26	0.018	0.16	Large
MEI	Group	6.00	0.020	0.16	Large
DM OC	Moment	4.37	0.045	0.12	Medium
DM mobility	Moment	4.81	0.036	0.13	Medium
DM space	Moment	13.19	<0.001	0.29	Large
DM offensive unit	Moment	14.29	<0.001	0.31	Large
DM DC	Moment	24.27	<0.001	0.43	Large
DM balance	Moment	10.60	0.0033	0.25	Large
DM defensive unit	Moment	61.81	<0.001	0.66	Large
DM defensive unit	Moment: Group	5.80	0.022	0.15	Large
DMI	Moment	8.58	0.006	0.21	Large
Performance	Moment	10.70	0.003	0.25	Large

LSST, Loughborough Football Shooting Test; MEI, motor efficiency index; DM, decision-making metric; DMI, decision-making index; *η*^2^, eta squared (effect size); qualitative ES, qualitative interpretation of effect size (small, medium, large).

The analysis examined the main effects of moment and group, as well as their interaction (moment × group) across multiple statistically significant variables are presented in Table 5. The findings revealed significant effects for several measures, highlighting differences between groups and changes over time. For duration (%), the main effect of moment was negligible (*F* = 4.53 × 10^−5^, *p* = 0.995, *η*^2^ = 6.57 × 10^−9^), as was the effect of group (*F* = 1.14 × 10^−6^, *p* = 0.999, *η*^2^ = 1.65 × 10^−10^). The moment × group interaction remained minimal (*F* = 3.10 × 10^−7^, *p* = 0.999, *η*^2^ = 4.50 × 10^−11^), suggesting that neither time nor group membership had a meaningful impact on this variable. For duration (ms), the main effect of moment was also negligible (*F* = 2.27 × 10^−5^, *p* = 0.996, *η*^2^ = 2.47 × 10^−9^), with group showing an even smaller effect (*F* = 2.68 × 10^−7^, *p* = 0.999, *η*^2^ = 2.92 × 10^−11^). The moment × group interaction was insignificant, indicating no meaningful variation between conditions. In the dwell time (fixation, %) variable, the group effect was notable (*F* = 6.27, *p* = 0.012, *η*^2^ = 0.001), suggesting that the groups differed significantly. However, the effects of moment (*F* = 1.57, *p* = 0.210, *η*^2^ = 0.0003) and the moment × group interaction (*F* = 0.003, *p* = 0.954, *η*^2^ = 5.70 × 10^−7^) were negligible, indicating that time and its interaction with group assignment had little influence. For TTFF (parent), the moment effect was negligible (*F* = 0.012, *p* = 0.912, *η*^2^ = 2.04 × 10^−6^), as was the group effect (*F* = 0.170, *p* = 0.680, *η*^2^ = 2.86 × 10^−5^). This suggests that there was no meaningful difference between groups or across time for this variable.

**Table 5:** Two-way ANOVA results comparing group and moment effects for eye-tracking variables.

Variable	Source of variation	*F*	*p*	*η* ^2^	Qualitative ES
Start	Moment	27.60	<0.001	0.003	Negligible
Parent start	Moment	20.66	<0.001	0.003	Negligible
Hit sequence	Group	11.29	<0.001	0.002	Negligible
Hit sequence	Moment	7.05	<0.001	0.001	Negligible
Hit sequence	Group: Moment	5.85	0.015612	0.001	Negligible
Last dwell duration	Group	4.55	0.032972	0.001	Negligible
Dwells with fixations	Group	13.52	<0.001	0.002	Negligible
Revisit count (fixation dwells)	Group	13.52	<0.001	0.002	Negligible
Fixation count	Group	14.62	<0.001	0.002	Negligible
TTFF sequence	Group: Moment	5.20	0.023	0.0009	Negligible
Dwell time (fixation, ms)	Group	5.65	0.017	0.0009	Negligible
Dwell time (fixation, %)	Group	6.27	0.012	0.001	Negligible
Duration of average fixation	Group	28.24	<0.001	0.005	Negligible
Duration of average fixation	Moment	3.91	0.048	0.001	Negligible
First fixation duration	Group	28.90	<0.001	0.005	Negligible
Last fixation duration	Group	13.98	<0.001	0.002	Negligible
Dispersion of average fixation	Group	19.25	<0.001	0.003	Negligible
Saccade count	Group	18.05	<0.001	0.005	Negligible
Duration of average saccade	Group	8.00	0.004	0.002	Negligible
Amplitude of average saccade	Group	39.98	<0.001	0.015	Small
Peak velocity of average saccade	Group	27.83	<0.001	0.008	Negligible
Peak acceleration of average saccade	Group	9.33	0.002	0.003	Negligible
Peak deceleration of average saccade	Group	26.36	<0.001	0.011	Small

TTFF, time to first fixation; AOI, area of interest. Eye-tracking variables include fixation count, duration, dispersion, dwell time, and saccade metrics (amplitude, velocity, acceleration, deceleration, direction) quantifying gaze efficiency. *η*^2^, eta squared (effect size).

## 4 Discussion

The primary objective of this study was to examine the impact of an intervention combining eye-tracking technology and neurofeedback training on the technical, tactical, and cognitive performance of female youth football players. It was hypothesized that integrating these technologies would lead to significant improvements in shooting accuracy, decision-making speed, and gaze efficiency, ultimately enhancing overall game performance. The results confirmed some aspects of this hypothesis, demonstrating notable enhancements in offensive decision-making and gaze behaviour, while physical performance outcomes did not improve to the same extent as cognitive and tactical measures. These findings suggest that while cognitive and visual processing can be improved through targeted interventions, physiological adaptations may require additional or different training approaches.

The study employed a combination of quantitative performance assessments, including eye-tracking metrics, decision-making indexes, and neurofeedback parameters, alongside technical and tactical evaluations. The validity of these methods is supported by their application in sports psychology and cognitive processing research, specifically in evaluating perceptual-motor skills and decision-making abilities (Casanova et al., 2022). Eye-tracking technology provides detailed data on gaze fixation and saccadic movements, which are crucial for understanding cognitive processes in sports contexts (Kooiker et al., 2016). Furthermore, neurofeedback tools, such as i-Brain, enhance the investigation of cognitive control and concentration, further informing how these cognitive factors influence athletic performance (Aksum et al., 2021; Casanova et al., 2022). The amalgamation of these quantitative and qualitative assessments deepens the understanding of cognitive and motor performance in athletic environments (Duchowski, 2013; Kooiker et al., 2016). The findings revealed significant improvements in several key areas. The MEI offensive unit demonstrated a substantial increase, suggesting that players in the experimental group developed better offensive positioning and movement efficiency. This aligns with findings by Scharfen and Memmert (2019), who confirm a strong connection between cognitive functions and effective sports performance, including the development of game intelligence, which is critical for offensive strategy execution. Similarly, DM balance and DM defensive unit showed large effect sizes, indicating enhanced decision-making stability and defensive coordination. Although Davis et al. does not specifically address decision-making effectiveness in athletic contexts (Almulla et al., 2020; Matos et al., 2023). Improvements in gaze-related metrics, such as fixation count and revisit count, suggest that participants became more efficient in visual search strategies, reinforcing the effectiveness of eye-tracking interventions echoing claims from Komarudin et al. (2021) and Mirwald et al. (2002). About the positive impact of cognitive training on women football players’ attentional focus during performance. The improvement in YYIR1 performance suggests that the intervention did not negatively affect intermittent recovery capacity, despite its primary cognitive focus. This finding reinforces the complexity of integrating cognitive and physical training, where increased cognitive load may interact with physical performance adaptations, a notion supported by Büchel et al. (2022), who note that increased cognitive demands can impair motor performance. These findings highlight the complexity of integrating cognitive and physical training in a cohesive framework (Teixeira et al., 2022b). A deeper physiological interpretation is warranted for the observed improvement in YYIR1 performance. Although the intervention primarily targeted cognitive processes, improvements in intermittent recovery capacity may reflect indirect benefits such as enhanced decision-making efficiency, pacing strategies, or neuromuscular coordination, as well as physical recovery capacity (Afonso et al., 2025a; Afonso et al., 2025b).

The results of this study revealed significant improvements in technical, tactical, and cognitive performance among female youth football players following the intervention. The MEI offensive unit exhibited a substantial increase, suggesting enhanced offensive positioning and movement efficiency. Similarly, DM defensive unit and DM balance showed large effect sizes, indicating improved defensive coordination and stability in decision-making. This is aligned with the results by Vestberg et al. (2017) that link executive functions to success in football. Eye-tracking measures demonstrated notable improvements, with dwell count (fixation) significantly increasing and revisit count (gaze dwells) significantly decreasing, reflecting more efficient gaze behaviours. This efficiency is crucial, as highlighted by Millard et al. (2022), noting the essential visual skills required for football that facilitate quick processing of information during match play. However, the YYIR1 test showed a significant improvement, suggesting that cognitive-perceptual training can be integrated without compromising aerobic performance, reinforcing the complexities of integrating cognitive and physical training preserving the physical demands (González-Víllora et al., 2022). The LSST shooting velocity displayed a significant group effect, indicating superior technical execution in the intervention group. The literature shows previous research, demonstrating technical skill development through targeted training strategies (Afonso et al., 2025a; Afonso et al., 2025b; Wang et al., 2023).

Neuroplastic mechanisms linking eye-tracking and EEG changes. Neurofeedback training harnesses neuroplasticity by enabling individuals to modulate their brain activity through real-time feedback; repeated practice facilitates reorganization of neural networks and improved self-regulation (Neurocare Group, 2025). The concurrent improvements observed in fixation efficiency (reduced revisits and greater dwell counts) and decision-making stability may reflect adaptations in the fronto-parietal networks that underpin attentional control and motor planning. The sensorimotor rhythm training targeted at C3, Cz and C4 likely strengthened corticospinal pathways, which, together with refined visual search strategies, could enhance anticipation and tactical awareness. Such interactions between neurofeedback-induced cortical plasticity and eye-movement behaviour have been hypothesized but rarely demonstrated in youth female athletes. Our findings thus provide preliminary evidence that coupling neurofeedback with gaze-training can promote neurocognitive adaptations beyond traditional physical drills.

This study has several strengths, including its novel application of combined eye-tracking and neurofeedback interventions in female youth football, a population often underrepresented in sports science research. Additionally, the methodological rigor, including controlled experimental conditions and validated performance measures, strengthens the reliability of the findings. However, some limitations should be acknowledged. The study did not account for potential external influences on physiological performance, such as weekly training volume, diet, or sleep quality. Furthermore, the intervention duration may not have been sufficient to elicit long-term physical adaptations. The exploratory cross-sectional design and small sample size (*n* = 8) limit the statistical power and generalizability of the findings, and all the results should be interpretated in an exploratory view and with caution. The participants were not randomized or compared against a control group, restricting causal inference. Inclusion and exclusion criteria were minimal (female football players aged 14–17 years with no history of neurological disorders), which may introduce selection bias. Nutrition and hydration, as well as body composition, were not monitored during the intervention, nor was body composition assessed; these factors could influence performance and should be controlled in future studies. Peak aerobic capacity (V̇O₂ max) was not measured, limiting conclusions about physiological training status. Fatigue was inferred from performance decline but was not objectively quantified through subjective scales or biomarkers. Finally, the heterogeneity of playing positions may have influenced tactical metrics; although statistical adjustments were attempted, larger randomized controlled trials are needed to confirm these preliminary observations. Future research should explore extended intervention periods, incorporate complementary physical training regimens, and examine individual variability in responsiveness to neurofeedback and gaze training. Expanding this research to different age groups and competitive levels will further contribute to a more comprehensive understanding of cognitive-motor interactions in youth women football performance.

Coaches and sport scientists aiming to replicate this protocol should integrate neurofeedback and eye-tracking sessions three times per week, each lasting approximately 30 min, over at least 6 weeks. These cognitive sessions should be scheduled on non-consecutive days and layered onto existing on-field and strength-training programmes rather than replacing them. Brief eye-tracking drills can be embedded within small-sided games to reinforce gaze efficiency without imposing excessive physiological load. Monitoring fatigue, hydration, and sleep quality is essential to mitigate potential declines in aerobic capacity, and sessions should be adjusted accordingly. We recommend a periodized approach in which neurofeedback is used during preparatory phases and reduced during competitive phases to balance cognitive load and physical conditioning.

## 5 Conclusion

This exploratory study provides preliminary evidence that the integration of eye-tracking and neurofeedback training can positively influence cognitive, technical, and tactical dimensions of performance in female youth football players. Specifically, improvements were observed in offensive motor efficiency (MEI offensive unit), defensive decision-making (DM defensive unit), and several gaze-related metrics, including fixation efficiency and reduced revisit behaviours. These findings suggest that targeted cognitive-perceptual training may enhance players’ ability to process visual information and make effective decisions during dynamic game situations, reinforcing the growing importance of neurocognitive factors in football performance.

From a physiological perspective, the observed improvement in YYIR1 performance indicates that the intervention did not negatively affect intermittent recovery capacity, despite its primary focus on cognitive and perceptual domains. This finding is particularly relevant, as it suggests that cognitive-based training strategies can be integrated into regular training routines without compromising aerobic fitness. Together, these results support the feasibility of combining traditional football training with technology-driven cognitive interventions to promote a more holistic development of young athletes.

However, these findings must be interpreted with caution due to several limitations inherent to the study design. The small sample size, absence of a control group, and exploratory nature of the intervention limit the generalizability and causal interpretation of the results. Additionally, key variables such as training load, nutrition, sleep, and fatigue were not controlled or directly measured. Future research should aim to validate these findings through larger randomized controlled trials, extended intervention periods, and integrated monitoring of physiological and cognitive adaptations. Despite these limitations, the present study offers a valuable proof of concept and contributes to the emerging evidence supporting the role of cognitive-perceptual training in youth football development.

## Data Availability

The raw data supporting the conclusions of this article will be made available by the authors, without undue reservation.
